# Free-breathing steady-state free precession 3D coronary MRA: investigation of the dependency on the running direction of the vessel and the direction of the motion correction

**DOI:** 10.1186/1532-429X-11-S1-P138

**Published:** 2009-01-28

**Authors:** Yuki Ohmoto, Rieko Ishimura, Takashi Yoshida, Yusuke Hamada, Yoshinori Tsuji, Junji Takahashi, Shigehide Kuhara, Sachiko Isono, Ayako Ninomiya, Hiroyuki Tsuji, Yasuji Arase, Shigeko Hara

**Affiliations:** 1 Toranomon Hospital Health management center, Minato-ku Tokyo, Japan; 2 Toranomon Hospital Cardiology, Minato-ku Tokyo, Japan; 3 Toranomon Hospital Radiology, Minato-ku Tokyo, Japan; 4 Toshiba Medical Systems, Otawara Tochigi, Japan

**Keywords:** Image Quality, Left Anterior Descend, Motion Correction, Toshiba Medical System, Coronary Magnetic Resonance Angiography

## Introduction

Coronary magnetic resonance angiography (CMRA) is a very useful and safe technique for the screening of coronary artery disease.

However, the image quality of CMRA depends on the individual performing examination and has some dependency on the direction of the vessel. The CMRA is performed under free breathing while monitoring the position of the diaphragm and the motion correction is performed mainly in the superior inferior (SI) direction. Therefore there may be some dependency on the running direction of the vessel and the direction of motion correction. Our preliminary study suggested that the image quality obtained with oblique saggital acquisition (SA) is better than that by axial acquisition (AX) for evaluating lesions in the LMT to the left anterior descending artery (LAD).

## Purpose

The purpose of this study was to evaluate 1) the image quality, 2) the coronary length that can be evaluated, and 3) the scan time, between image acquisition by AX and SA.

## Methods

Eleven healthy volunteers (7 men, 4 women, mean age; 38 ± 11 years) underwent CMRA at 1.5 T (Toshiba Medical Systems Excelart Vantage™ powered by Atlas) using a16-channel phased-array coil. The elements were arranged four-by-four at both the front and back, and the two element rows were used to cover the whole heart. Imaging was performed by applying Real-Time Motion Correction (RMC) method to compensate the respiratory motion and corrected cardiac-triggered steady-state-free-precision (SSFP) sequence with fat suppression and T2 preparation. The imaging parameters were: TR/TE/FA = 4.3 ms/2.2 ms/120°, spatial resolution = 1.5 × 1.5 × 1.5 mm^3^. The 2D parallel imaging was applied with in phase and slice directions by a factor of 2.1 and 1.4. The data of the CMRA was transferred to the workstation with image reconstruction software (AZE VirtualPlace Fujin, AZE Ltd., Tokyo, Japan). The differences in the image quality were assessed by two experienced observers using randomized image pairs (four-point scale: 1 = poor, 2 = moderate, 3 = good, 4 = excellent). Motion artifacts and coronary delineations were used as criteria in the scoring of the quality. We also evaluated the available coronary lengths, that could be given a score of more than 2 by visual estimation, and the scan time.

## Results

**Figure 1:**
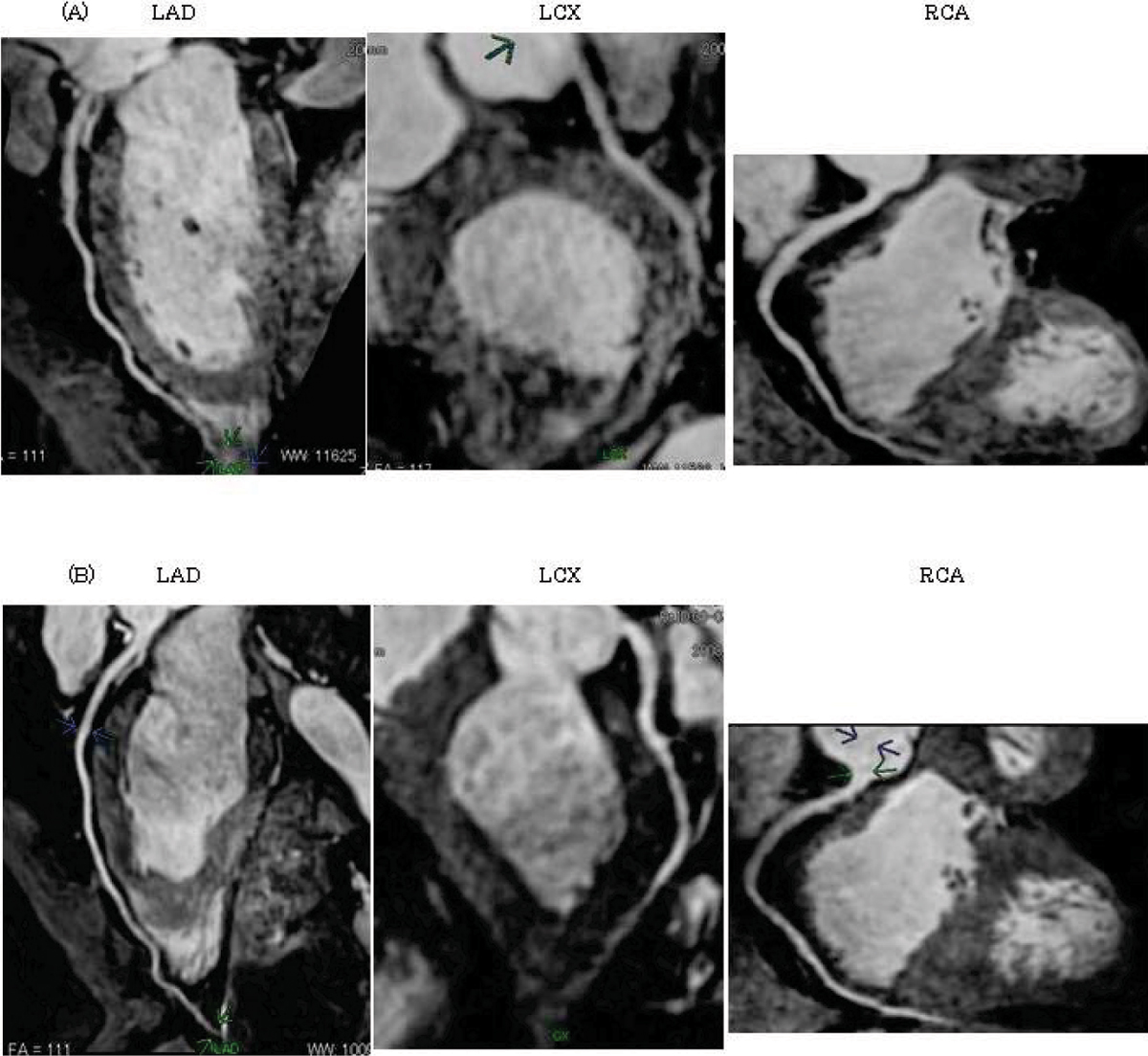
**The images obtained by axial acquisition (A) and oblique sagittal acquisition (B)**.

The image qualities of coronary arteries between AX and SA were 3.5 ± 0.5 and 2.9 ± 0.3 for RCA (p < 0.05), 2.4 ± 0.7 and 3.6 ± 0.5 for LMT (p < 0.01), 2.6 ± 0.7 and 3.5 ± 0.5 for LAD (p < 0.01), 2.3 ± 0.9 and 3.2 ± 0.9 for LCX (p < 0.01) in each proximal lesion. The coronary lengths evaluable in the images acquired by AX and SA were 136.4 ± 42.0 and 139.6 ± 41.7 mm for RCA (n.s.), 115.2 ± 26.0 and 124.4 ± 25.4 mm for LAD (n.s.), and 79.5 ± 30.5 and 95.9 ± 24.7 mm for LCX(p < 0.01). The scan time for image acquisition by AX and SA were 680 ± 159 and 763 ± 177 seconds (p < 0.05), respectively. Figure 1.

## Conclusion

SA provides comparatively better image quality of coronary lesions in some arterial segments, especially the LMT and LAD. Although other lesions were not depicted as clearly as in the images acquired by AX, SA provided valuable diagnostic information on lesions of the LMT. We suggest that SA and AX may be used to evaluate the coronary arteries in a complementary manner.

